# Open versus closed reduction internal fixation for lateral condyle humeral fractures in children: a systematic review and meta-analysis

**DOI:** 10.1186/s13018-023-03808-3

**Published:** 2023-04-26

**Authors:** Suyue Zhu, Yan Zheng, Yazhou Jiang, Hanjun Yin, Dongsheng Zhu

**Affiliations:** 1 Department of Pediatrics, Suqian Hospital Affiliated to Xuzhou Medical University, Suqian, China; 2 Department of Pediatric Orthopedics, The First People’s Hospital of Lianyungang, Lianyungang, 222000 Jiangsu Province China

**Keywords:** Closed reduction, Lateral condyle humeral fractures, Meta-analysis

## Abstract

**Objective:**

The objective of this meta-analysis was to illustrate the clinical outcomes and safety of two different management options for Song stage 2–4 lateral condyle humeral fractures in children.

**Method:**

In January 2023, a systematic computer-based search was conducted. Data were retrieved for patients with two different management options for lateral condyle humeral fractures in children. The primary endpoints were clinical outcomes based on infection, avascular necrosis, and nonunion. After testing for publication bias and heterogeneity between studies, the data was aggregated for stochastic effect models when necessary.

**Results:**

Eight clinical studies with 742 patients were eventually included in the meta-analysis. There was no significant difference between the closed reduction and percutaneous pinning, and open reduction and internal fixation in terms of the clinical outcomes based on infection, avascular necrosis, and nonunion (*P* > 0.05).

**Conclusions:**

Closed reduction and percutaneous pinning, as well as open reduction and internal fixation of lateral condyle humeral fractures in children, resulted in similar structural stability and functional outcomes. More high-quality randomized controlled trials are needed to determine this conclusion.

## Introduction

Lateral humeral condyle fracture is the second most common elbow fracture in the pediatric age group, after supracondylar fractures. These fractures are peculiar in that they are intra-articular and are prone to displacement due to the attachment of the extensor muscles of the forearm to the lateral condyle. Because this, if not managed properly, they can cause a variety complications including elbow deformity [1]. Traditionally, open reduction and internal fixation (ORIF) was preferred in order to assure anatomic reduction of this physeal, intra-articular fracture [2]. In the last decade, there has been renewed interest in alternative approaches, such as closed reduction and percutaneous pinning (CRPP) for Song stage 2–4 lateral condyle humeral fractures [3]. Until now, there has been no general agreement among orthopedic surgeons on the most appropriate treatment for lateral condyle fracture of the humerus. Currently, there is no relevant meta-analysis comparing closed versus open reduction for lateral condyle humeral fractures in children.

It is therefore necessary to evaluate the efficacy and safety of two different management options for the treatment of Song stage 2–4 lateral condyle humeral fractures in children. This meta-analysis was designed to illustrate the clinical outcomes and safety of two different management options for Song stage 2–4 lateral condyle humeral fractures in children. We hypothesize that CRPP and ORIF have similar clinical outcomes for lateral condyle humeral fractures in children.

## Methods

This study was conducted in accordance with the guidelines of the revised assessment of multiple systematic reviews and the preferred reporting items of the systematic reviews and meta-analyses 2020 statement [4].

### Search strategies

Electronic searches were performed between 2012 and 2022 using EMBASE, PubMed, and MEDLINE. The search terms lateral condyle humeral fracture, open reduction and internal fixation, closed reduction and percutaneous pinning, and children were used individually and in combination. References, reviews and meta-analyses were then scanned for additional articles. We also performed a search on Google Scholar to review the references of selected studies.

### Study selection

Children with radiographically confirmed lateral condyle humeral fractures who has received ORIF or CRPP were recruited. Inclusion criteria: (1) clinical trials that compared ORIF versus CRPP between 2012 and 2022; (2) children younger than 18 years with lateral condyle humeral fractures; (3) original data included some of the following: operative time, infection, avascular necrosis, and nonunion. Exclusion criteria: (1) lateral condyle humeral fractures with other fractures, (2) pathological fractures, (3) case reports, cadaver or model studies, and biomechanical studies; (4) duplicate publications or studies did not provide sufficient raw data.

### Data extraction

Two authors independently extracted data from the list of the included studies, and a third reviewer was required to make a final determination in the event of any discrepancies. The objective of our analysis was to assess the operative time, postoperative complications including infection, avascular necrosis, and nonunion.

### Statistical analysis

Review Manager Software 5.3 was used for statistical analysis of the data. For the continuous variables, we computed the mean differences with 95% confidence intervals. For dichotomous variables, we used odds ratios. Statistical Heterogeneity was assessed using the Chi square test with significance set at *P* < 0.10. If the *I*^2^ value was less than 50%, a fixed-effects approach was applied; if the *I*^2^ value was 50% or more, then a random effect approximation was performed instead of a fixed-effect analysis.

## Results

### Search results and quality assessment

**Fig. 1:**
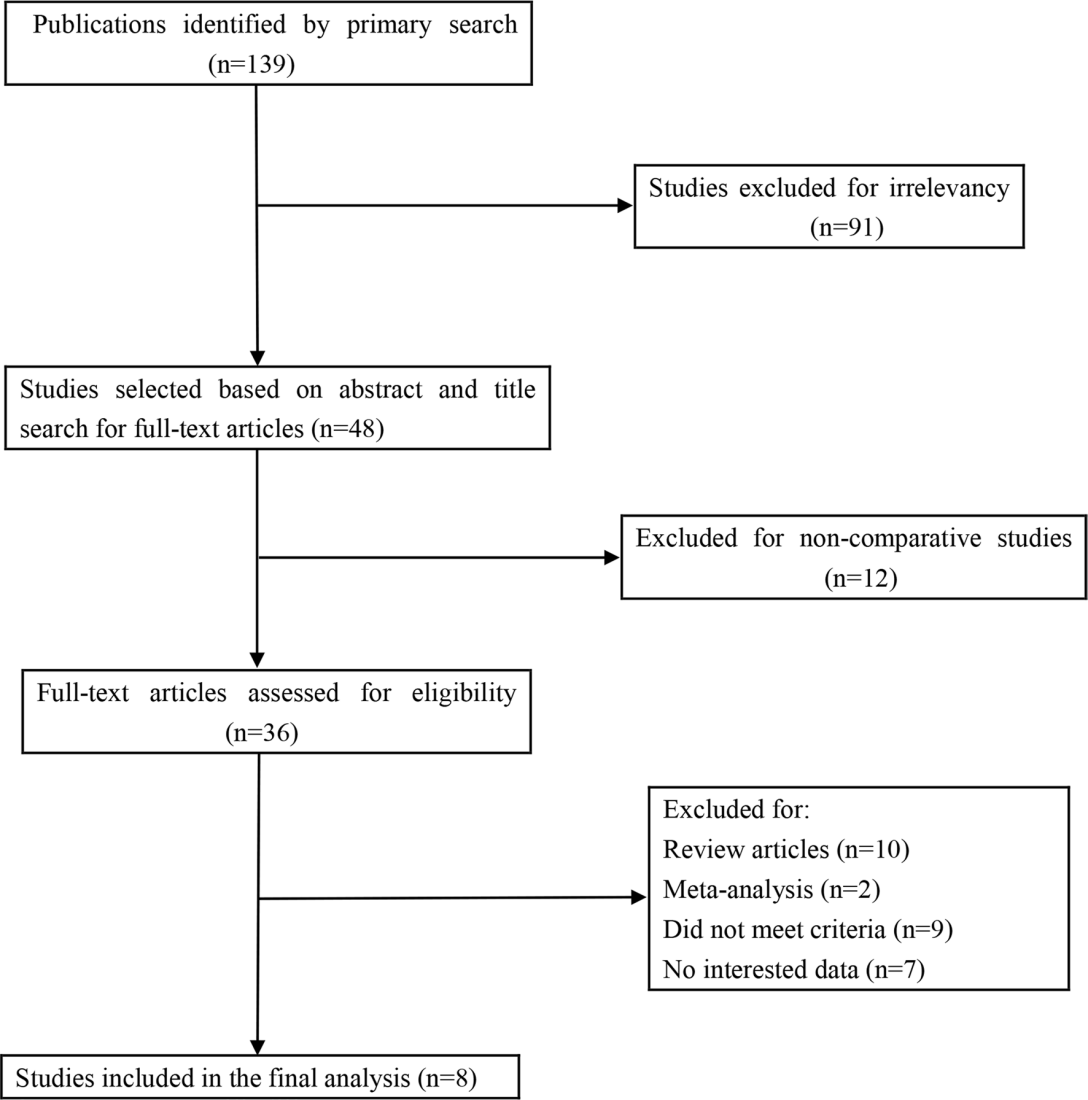
Diagram of workflow in the systematic review and meta-analysis

The initial search strategy identified 139 citations, of which 48 of them were retrieved for full text review. Eight trials met total inclusion criteria (Fig. 1): Silva et al. [3], Pennock et al. [5], Justus et al. [6], Gendi et al. [7], Nazareth et al. [8], Xie et al. [9], Xie et al. [10], Weng et al. [11]. The eight studies selected included 742 participants. Of these pediatric patients, 210 received CRPP and 532 received ORIF. Table 1 outlined the basic demographic data for each of the included studies, including study style, and age for each group. Table 2 displays the results obtained from each study.

**Table 1:** Demographic data, such as country, surgical type and age (years)

References	Country	Group		Age (years)	
		CRPP	ORIF	CRPP	ORIF
Silva et al. [3]	USA	28	163	NR	NR
Pennock et al. [5]	USA	23	51	NR	NR
Justus et al. [6]	USA	31	141	5.16 ± 2.15	5.29 ± 2.27
Gendi et al. [7]	USA	28	41	5.16	6.0
Nazareth et al. [8]	USA	8	30	5.8 ± 1.5	5.8 ± 1.9
Xie et al. [9]	China	45	62	5.3 ± 2.5	5.1 ± 2.2
Xie et al. [10]	China	37	13	NR	NR
Weng et al. [11]	China	10	31	4.90 ± 2.33	5.39 ± 2.03

*NR* not reported, *CRPP* closed reduction and percutaneous pinning, *ORIF* open reduction and internal fixation

**Table 2:** Operation time, infection, avascular necrosis, nonunion, and overall complications in CRPP and ORIF

References	Operation time		Infection		Avascular necrosis		Nonunion		Overall complication	
	CRPP	ORIF	CRPP	ORIF	CRPP	ORIF	CRPP	ORIF	CRPP	ORIF
Silva et al. [3]	25.4 (18–50)	52.6 (24–121)	1	3	0	5	0	2	1	10
Pennock et al. [5]	NR	NR	1	3	0	3	0	1	3	13
Justus et al. [6]	NR	NR	2	11	NR	NR	0	1	2	12
Gendi et al. [7]	78.64	123.29	0	6	3	5	2	0	7	11
Nazareth et al. [8]	NR	NR	1	1	0	0	1	3	2	4
Xie et al. [9]	38.8 ± 11.8	49.7 ± 7.2	0	0	0	0	0	0	NR	NR
Xie et al. [10]	NR	NR	0	0	0	0	0	0	5	3
Weng et al. [11]	36.00 ± 9.16	56.1 ± 9.99	0	6	0	0	0	0	7	11

*NR* not reported, *CRPP* closed reduction and percutaneous pinning, *ORIF* open reduction and internal fixation

### Operative time

**Fig. 2:**
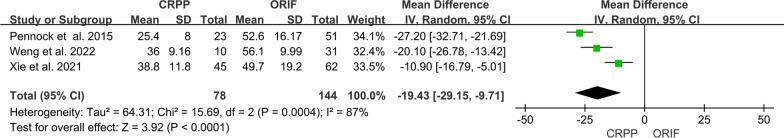
Forest plot comparing operation time for children treated with CRPP versus ORIF

Operational times were reported in three studies [5, 9, 11]. The meta-analysis demonstrated statistically significant difference in mean operating time for CRPP compared with ORIF (95% CI − 29.15 to − 9.71; *P* < 0.0001) (Fig. 2). There was evidence of significant statistical heterogeneity (*I*^2^ = 87%).

### Infection

**Fig. 3:**
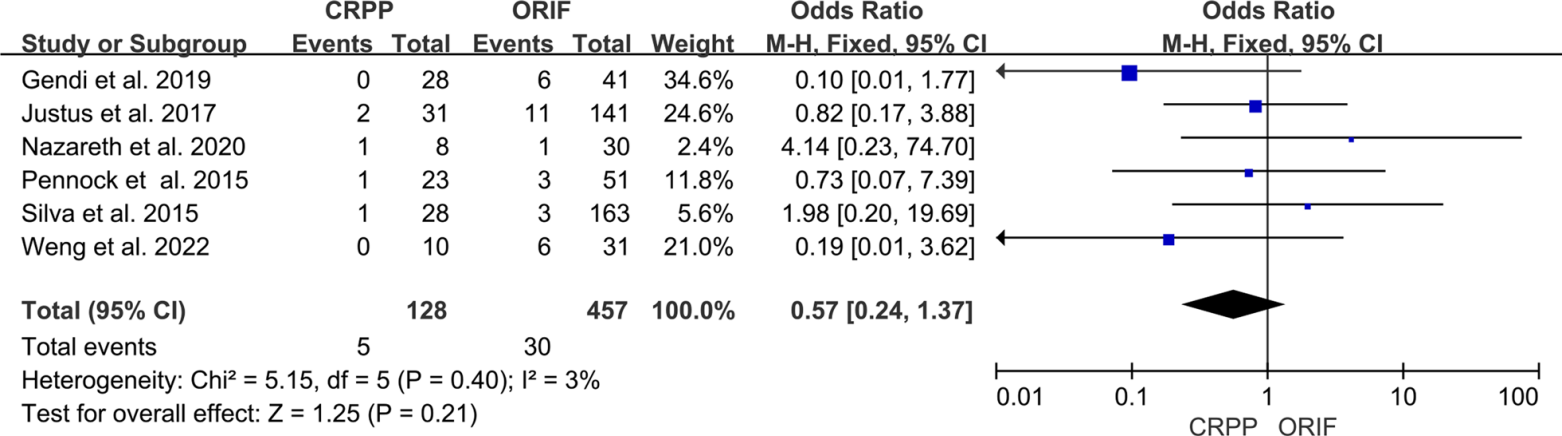
Forest plot comparing infection for children treated with CRPP versus ORIF

The incidence of postoperative infection was reported in six studies [3, 5–8, 11]. There were 5 infection (3.91%) in the CRPP group and 30 (6.56%) in the ORIF group. The meta-analysis demonstrated no statistically significant difference between the groups (95% CI 0.24–1.37; *P* = 0.21) (Fig. 3). There was no evidence of statistical heterogeneity (*I*^2^ = 3%).

### Avascular necrosis

**Fig. 4:**
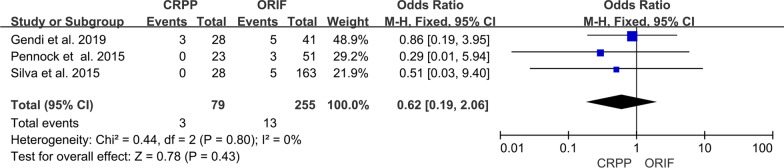
Forest plot comparing avascular necrosis for children treated with CRPP versus ORIF

Rates of avascular necrosis were reported in three studies [3, 5, 7]. There were 3 cases of avascular necrosis (3.79%) in the CRPP group and 13 (5.09%) in the ORIF group. The meta-analysis demonstrated that three was no statistically significant difference between each group (95% CI 0.19–2.06; *P* = 0.43) (Fig. 4). There was no evidence of statistical heterogeneity (*I*^2^ = 0%).

### Nonunion

**Fig. 5:**
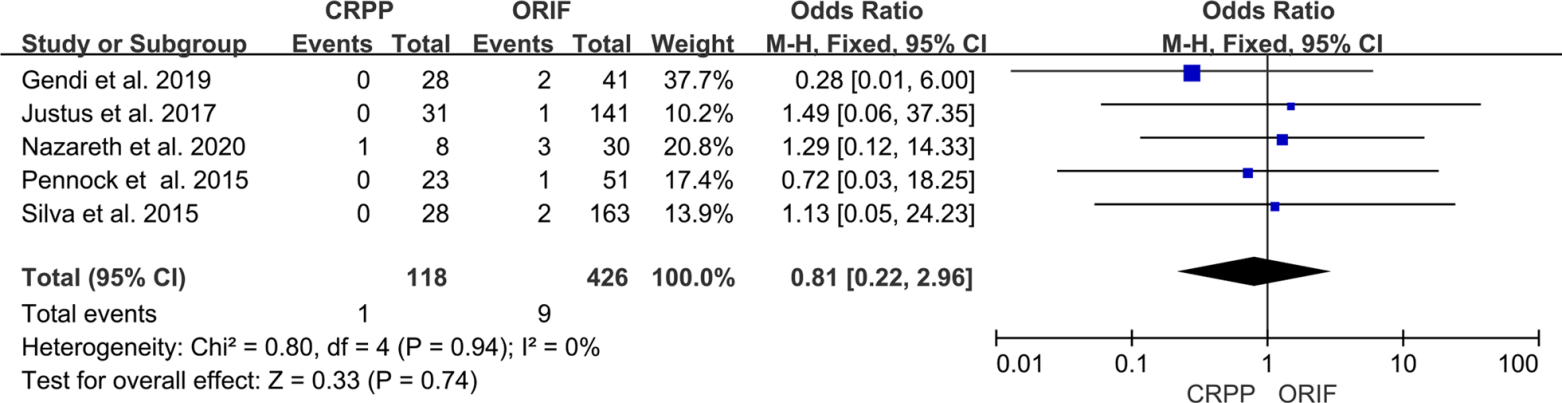
Forest plot comparing nonunion for children treated with CRPP versus ORIF

We extracted data on the incidence of nonunion in three studies [3, 5–8]. Nonunion was reported in 1 out of 118 patients in the CRPP group and 9 out of 428 patients in the ORIF group. The pooled analysis showed no difference between the two groups in the incidence of nonunion (95% CI 0.22–2.96; *P* = 0.74) (Fig. 5). Heterogeneity was considered to be low (*I*^2^ = 0%).

### Overall complication

**Fig. 6:**
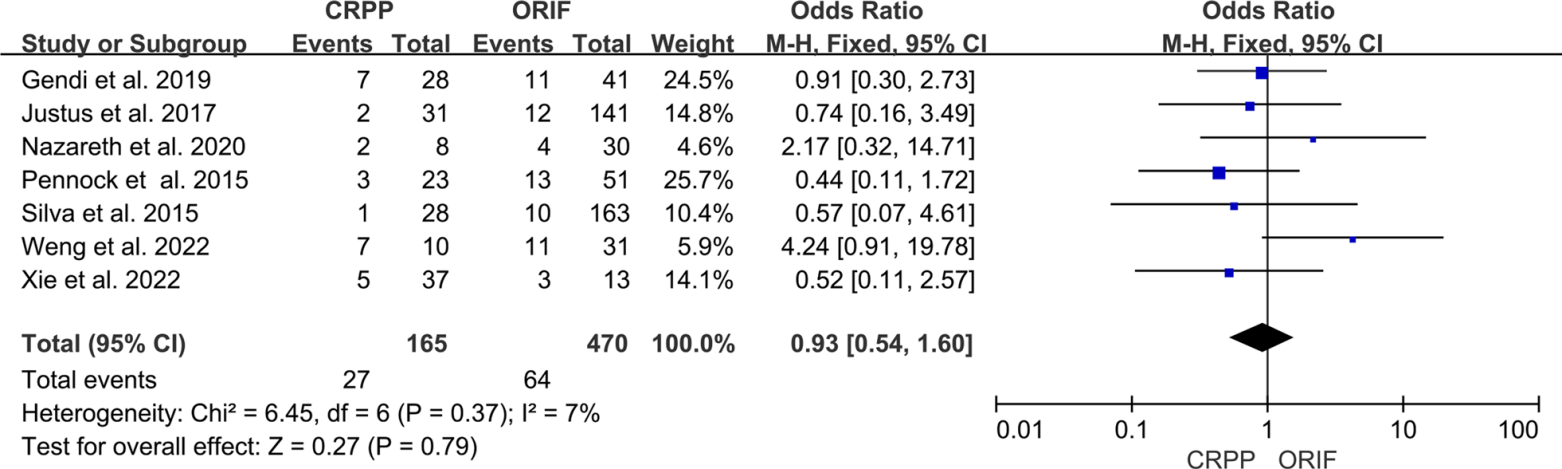
Forest plot comparing overall complication for children treated with CRPP versus ORIF

The incidence of complications was reported in seven studies [3, 5–8, 10, 11]. It was reported in 27 of 165 patients in the CRPP group and 64 of 470 patients in the ORIF group. The pooled analysis showed no difference between the two groups in overall complication rates (95% CI 0.54–1.60; *P* = 0.79) (Fig. 6). Heterogeneity was considered to be low (*I*^2^ = 7%).

## Discussion

This is the first systematic review and meta-analysis comparing different management options for lateral condyle humeral fractures in children. Results were based on outcomes such as infection, avascular necrosis, and nonunion. Results showed that there was no significant difference between the two groups regarding the aforementioned outcomes.

Because lateral condyle fractures are generally intra-articular, the ideal treatment for Song stage 2–4 lateral condyle humeral fractures is, according to many authors, CRPP [2]. In the current study, we found that CRPP had comparable clinical outcomes in terms of overall complications. Restoration of the articular surface and internal fixation is central to the prevention of malunion and nonunion in pediatric humeral lateral condylar fractures, and thus ORIF is recommend by most scholars [2, 12]. Previously, some studies initially tried CRPP for lateral condylar humeral fractures, followed by open reduction if closed reduction failed and the complications were possible [9, 13]. As a result, the open reduction group generally included the more complicated patients and the clinical outcomes were consistently worse than in the closed reduction group [9]. Therefore, several scholars have recently reported the implementation of CRPP for lateral condylar humeral fractures [14, 15]. In the findings of this meta-analysis, we found no significant differences in overall complication.

Infection was the most common complication after a lateral condyle humeral fracture. According to a systematic review of pediatric lateral condylar fractures, the incidence of infection has been reported as 4.8% [16]. In the current meta-analysis, infection rates were 3.91% and 6.56% for CRPP and ORIF, respectively. There was no significant difference between the two groups (*P* = 0.21). These rates were also comparable to those reported in previous literatures. Alexander et.al revealed that there was no significant difference between the open group and closed group in terms of the pin tract infection [8].

Growth arrest can occur when ossification nucleus is involved [17]. Avascular necrosis was reported to develop in 1.7% of cases [16]. It was previously attributed to a medial instability during fracture fusion, which caused separation between the trochlear and entrapped epiphyseal plates, while interference with physis vascular supply led to vascular necrosis [18]. Milch Type 2 fractures, Jakob Type 3 fractures, displaced fractures and delayed treatment increased the risk of avascular necrosis [19, 20]. It is relevant to mention that lag screw osteosynthesis appears to be associated with a reduced risk of avascular necrosis [19]. In the current meta-analysis, avascular necrosis occurred in 3.79% of CRPP and 5.09% of ORIF patients, respectively. There was no significant difference between the two groups.

Most of the lateral condyle humeral fractures had a uneventful union with a mean duration of 6.4 weeks and had a mean duration of 5.6 weeks for wire removal [21]. However, nonunion appeared to be more common in lateral condyle fractures than in other elbow fractures. Previous studies have put the risk at between 1 and 5%, depending on the definition used [22, 23]. The only treatment option for nonunion of the lateral condyle humeral is operative intervention. Surgical management for nonunion includes a variety of combinations of neurolysis and anterior transposition of ulnar nerve, corrective humeral osteotomy and osteosynthesis [24]. In the current meta-analysis, the rate of nonunion was 0.84% for CRPP and 2.11% for ORIF, respectively. There was no significant difference between the two groups.

The meta-analysis showed that CRPP was associated with shorter operative time in children (*P* < 0.0001).Taken together, in the surgical treatment of lateral condylar humerus fractures, CRPP led to shorter operative time and no increase in related complications such as infection, avascular necrosis, and nonunion compared to ORIF. Both CRPP and ORIF can achieve satisfactory clinical outcomes in the treatment of Song stage 2–4 lateral condylar humerus fractures. No differences in overall complications or prognosis were found between the two groups. However, CRPP has shown some advantages over ORIF, such as less invasive surgery, and shorter operation time.

There were several limitations to this meta-analysis: (1) no randomized clinical trials were available in the literature; (2) only 8 potential studies were eventually included, the effect size was relative small; (3) the internal fixation was different and, therefore, may led to heterogeneity in outcomes; (4) no follow-up and clinical score to assess the patient's activities.

## Conclusion

In summary, the CRPP and ORIF of Song stage 2–4 lateral condylar humeral fractures in children result in similar structural stability and functional outcomes. In terms of complications, there was no significant difference between the two administrations. Due to the limited sample size and the number of included studies, a multi-center RCT was required to identify the effects of closed reduction and percutaneous pinning for Song stage 2–4 transverse condylar humeral fractures in children.

## Data Availability

Datasets are available through the corresponding author upon reasonable request.
